# Supplementary figures and images for: Metagenomic Insights into Disease-Induced Microbial Dysbiosis and Elemental Cycling Alterations in Morchella Cultivation Soils: Evidence from Two Distinct Regions
Source: J Fungi (Basel). 2025 Sep 10;11(9):663. doi: 10.3390/jof11090663 (PMC12470759; doi:10.3390/jof11090663)

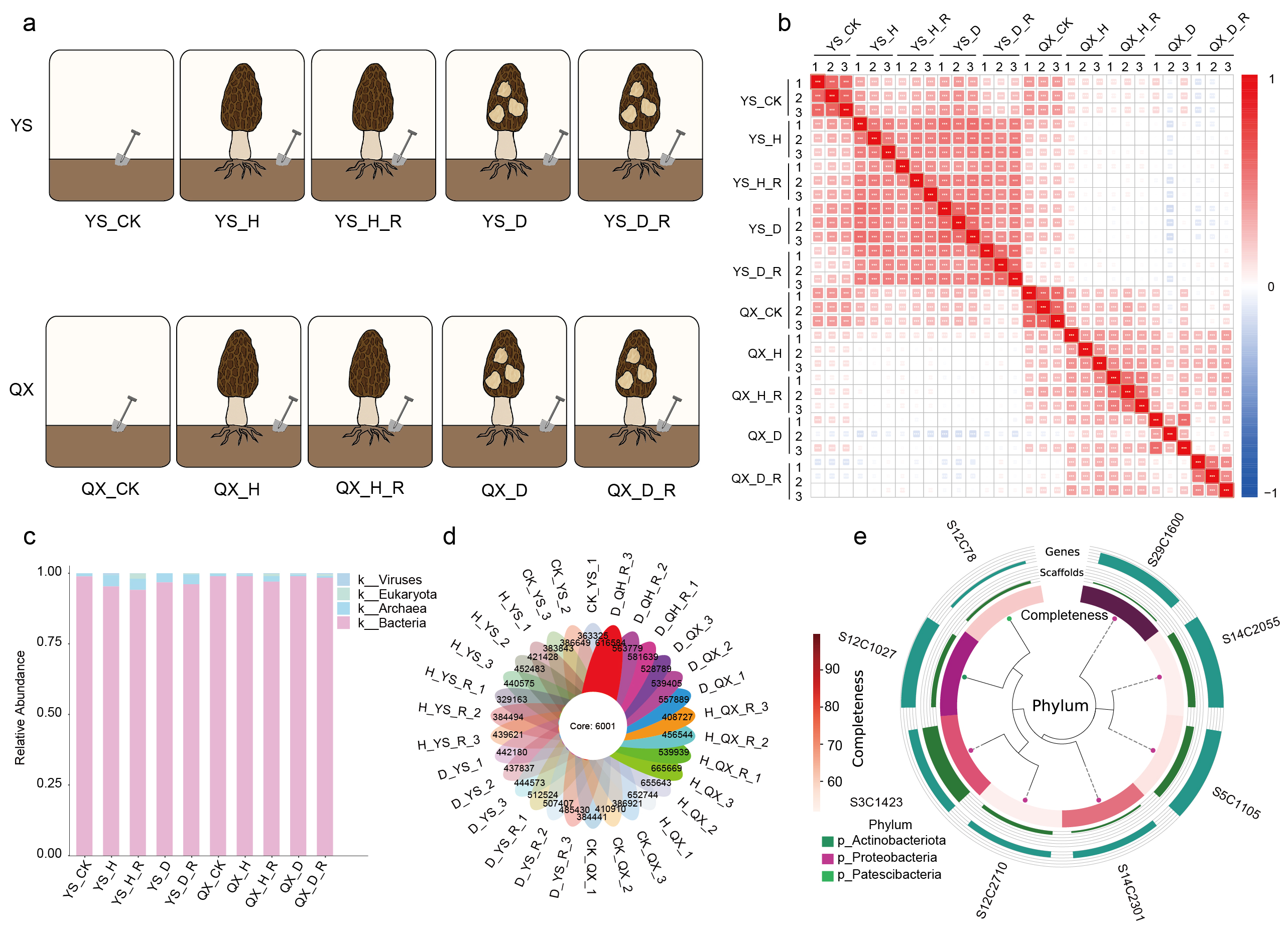

Supplement: Supplementary file 1 [file jof-11-00663-s001.zip › Figures/Figure 1.jpg]

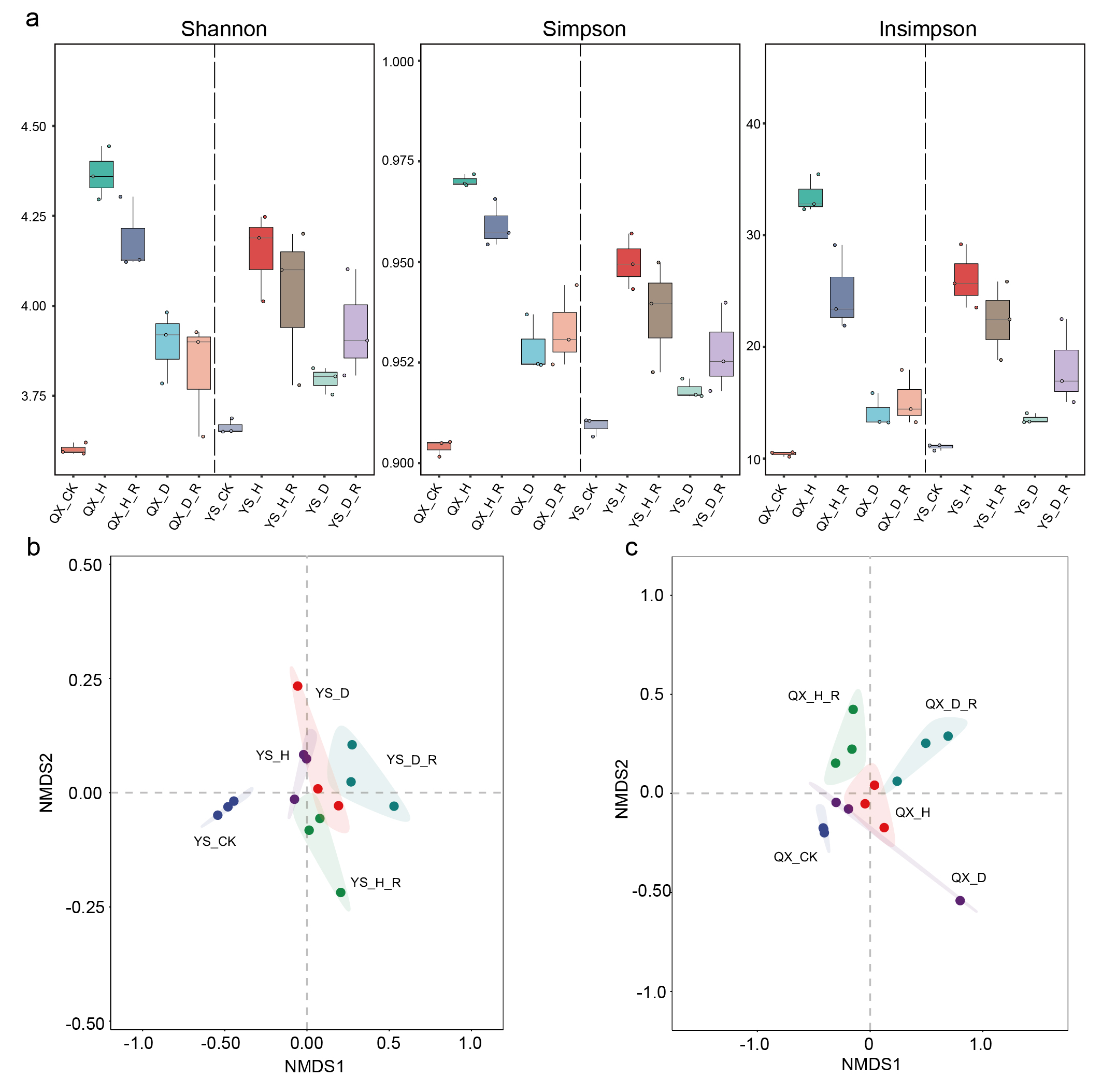

Supplement: Supplementary file 1 [file jof-11-00663-s001.zip › Figures/Figure 2.jpg]

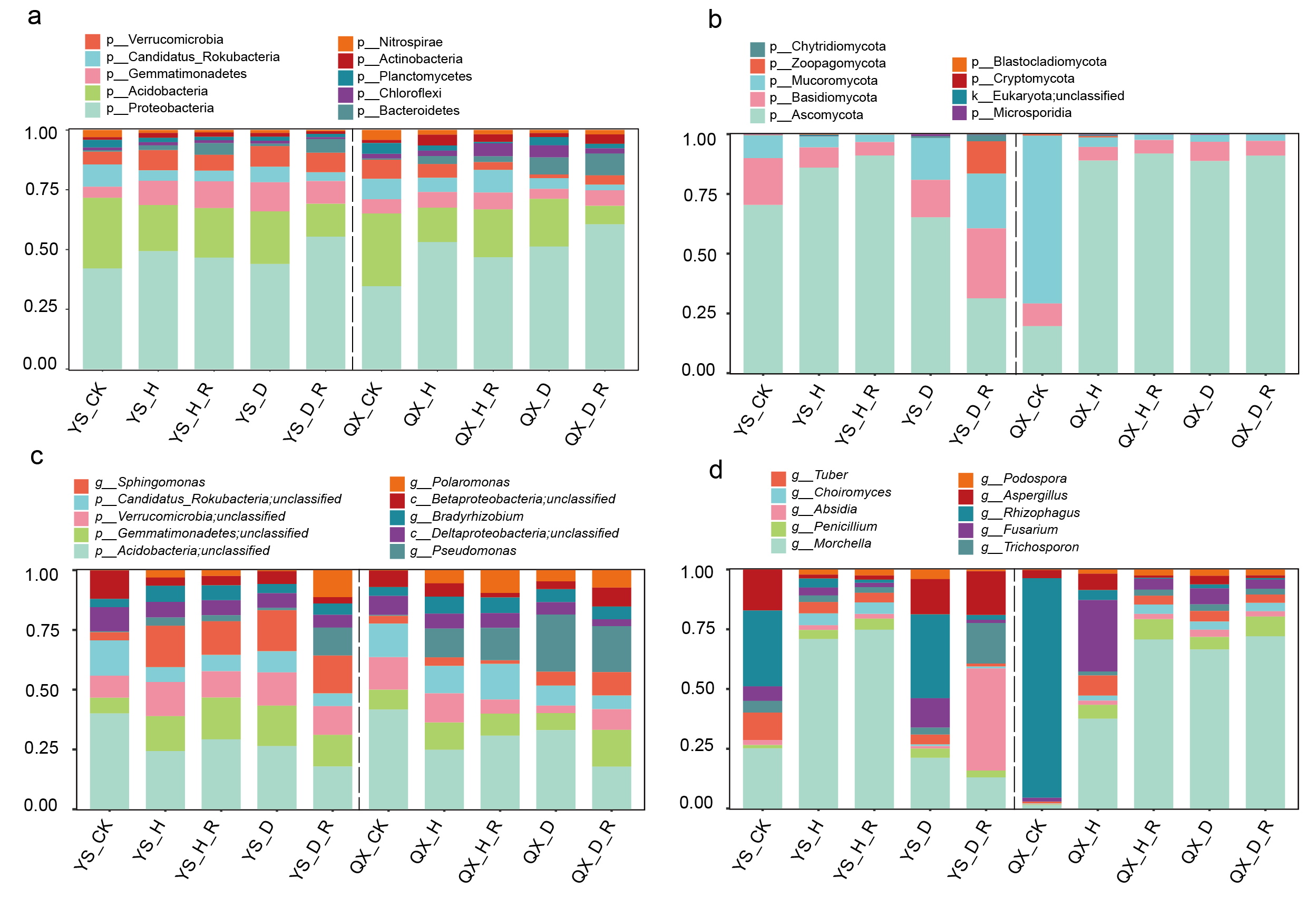

Supplement: Supplementary file 1 [file jof-11-00663-s001.zip › Figures/Figure 3.jpg]

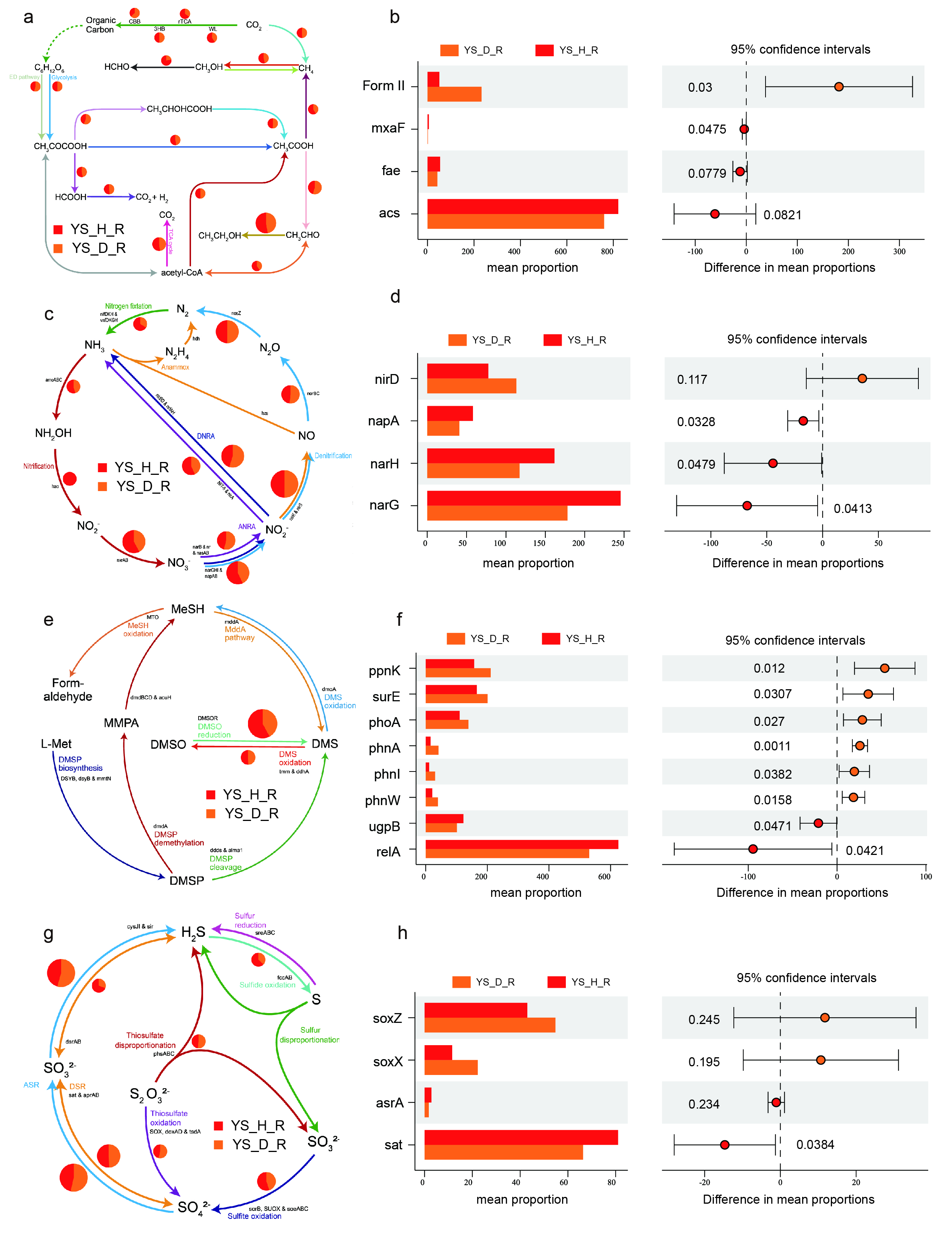

Supplement: Supplementary file 1 [file jof-11-00663-s001.zip › Figures/Figure 7.jpg]

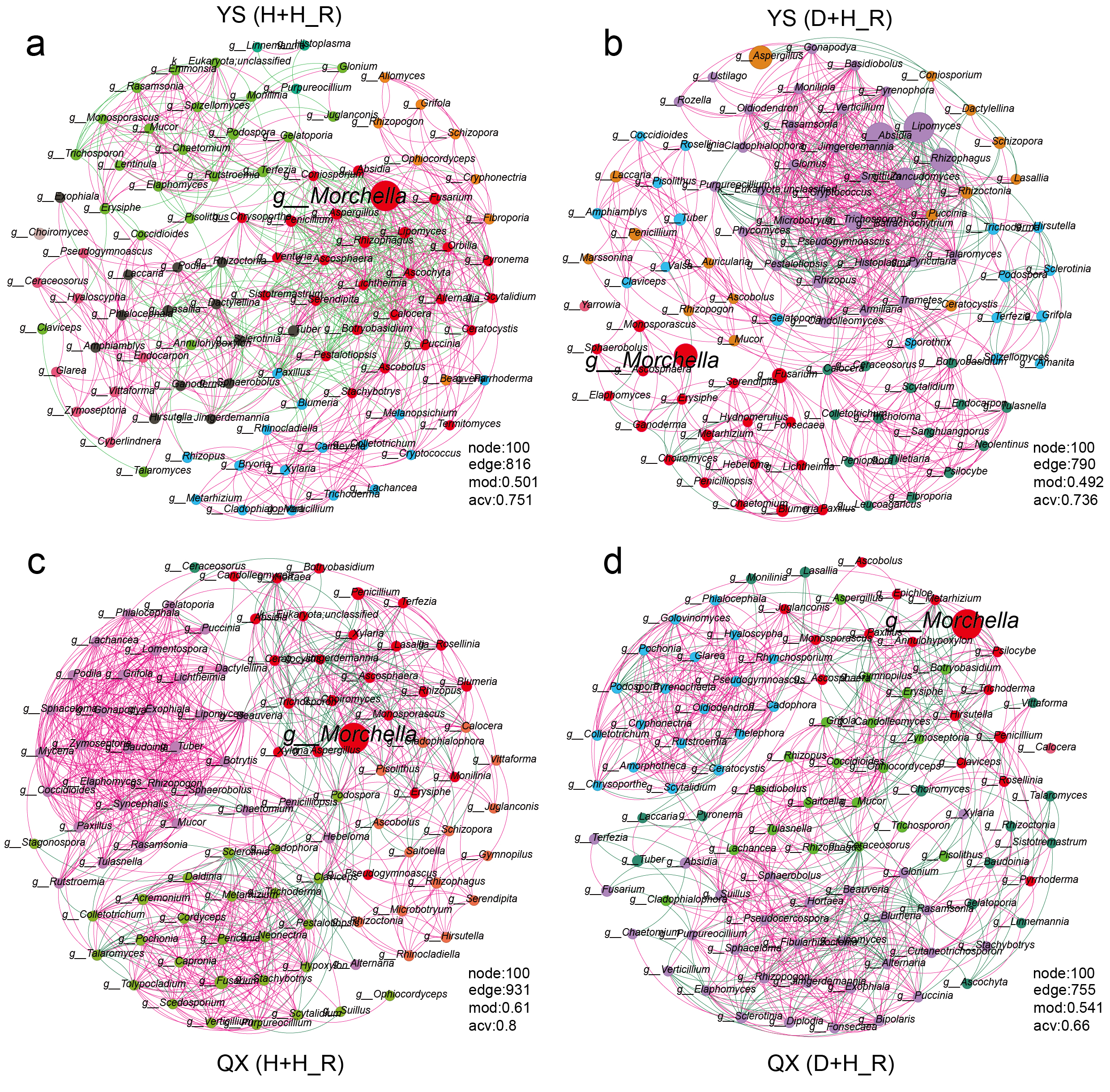

Supplement: Supplementary file 1 [file jof-11-00663-s001.zip › Figures/Figure 6.jpg]

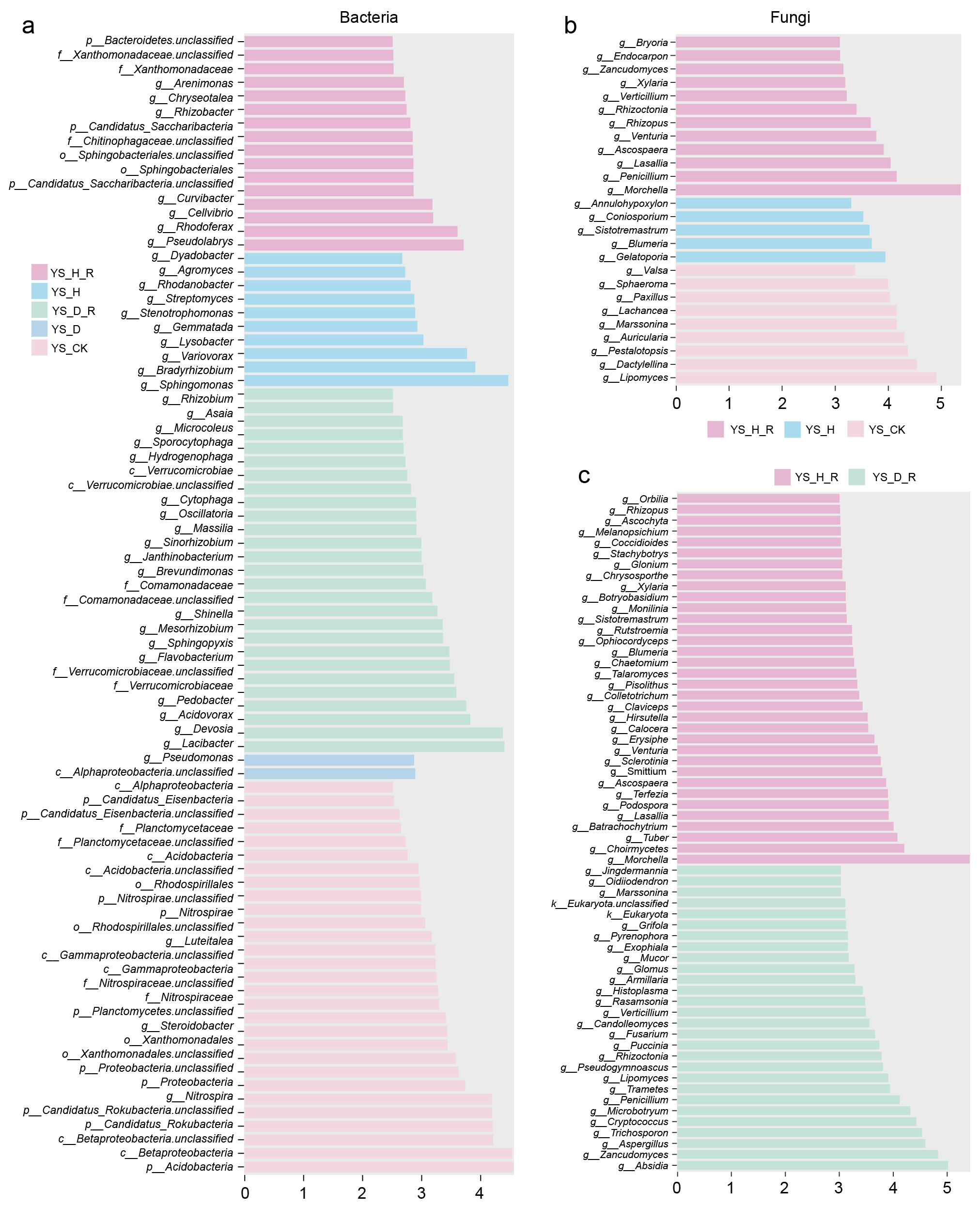

Supplement: Supplementary file 1 [file jof-11-00663-s001.zip › Figures/Figure 4.jpg]

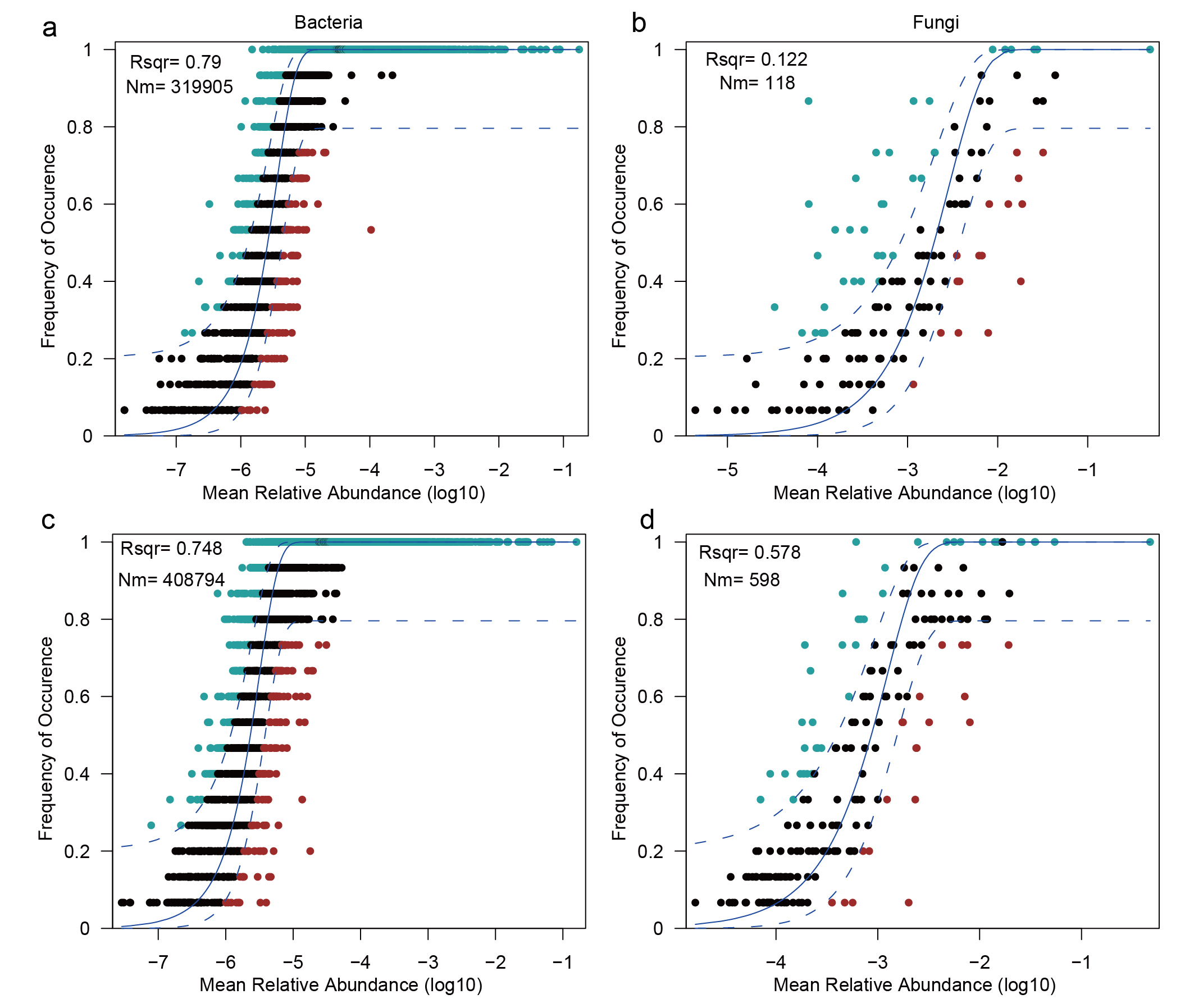

Supplement: Supplementary file 1 [file jof-11-00663-s001.zip › Figures/Figure 5.jpg]

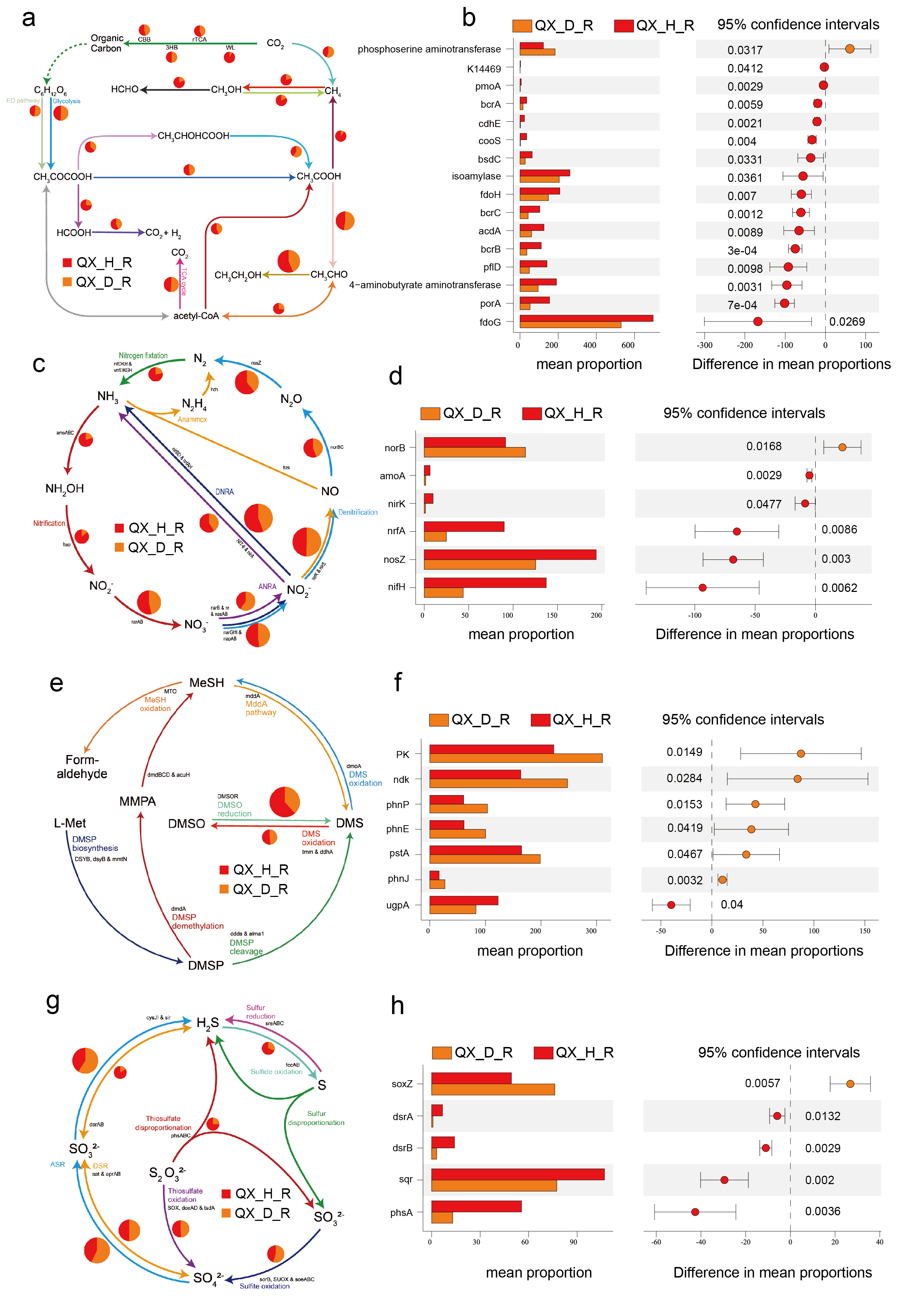

Supplement: Supplementary file 1 [file jof-11-00663-s001.zip › Figures/Figure 8.jpg]
